# 

**Published:** 1893-07-22

**Authors:** 


**OUR NEW OFFICES**

428, Strand, W.C., will henceforth be the Office of this Journal.

**Notices of Births, Deaths, and Marriages**, are inserted in this column at a charge of 2s. 6d. for an announcement not exceeding four lines.

### NOTICE TO CORRESPONDENTS.

All MS., Letters, Books for Review, and other matters intended for the Editor should be addressed The Editor, The Lodge, Porchester Square, London, W.

The Editor cannot undertake to return rejected M.S., even when accompanied by stamped directed envelope.

**Notice to Advertisers and Subscribers.**

All Advertisements, Orders for Copies of The Hospital, and Business Communications should be addressed to The Publisher, *not to the Editor*, The Hospital, 428, Strand, W.C.

The Cost of Subscription, post free, is as follows:—Per week, 2½d.; per quarter, 2s. 6d.; per half-year, 5s.; per annum, 8s. 6d. Foreign:—Per annum, 10s. 8d.; payable, in all cases, in advance.

**"Burdett's Hospital Annual," 1893.**

Fourth year of publication. 700 pp. Boards, gilt lettering. A most complete guide to British, Colonial, Indian, and American Hospitals, Dispensaries, Nursing and Convalescent Institutions, and Asylums. Price 5s. Published by The Scientific Press (Limited), 428, Strand, W.C.

**NOTICE.—The Index for Vol. XIII. (ending March 1893) is on sale at the Office of this Journal, price 2½d., post free.**

### SCRAPS AND GLEANINGS.

SERIOUS outbreaks of scarlet fever and small-pox are still reported from Leicester.

THE Free Eye and Ear Hospital at Southampton wish to go into larger and more convenient premises.

THIS year the amount collected at the Southampton Hospital Sunday Fund was £1,105, being an increase on the previous year's collection.

AN epidemic of measles has been raging at Dumbarton, and the need for a commodious and properly equipped hospital for infectious diseases has been convincingly brought home to the residents in its vicinity.

MRS. PITMAN, a lady whose name is closely associated with charitable works in Cheltenham, has contributed a sum of £1,350 to an institution called the Cotswold Convalescent Home, shortly to be inaugurated for that town.

THE death-rate of the Jewish peasants in Russia is abnormally high, reaching in some provinces the rate of 62 in 1,000. A recent congress of Russian physicians explained the reason to be insufficiency of nourishment.

THE Totnes Cottage Hospital, which is a very deserving institution, supported by voluntary contributions, was benefited this month by the proceeds of a grand fancy bazaar, held in the grounds of the Duke of Somerset's estate.

THE sewage farms in Berlin are in no way to be held accountable, Professor Virchow declares, for any cases of typhoid fever in that city. A discussion on the subject was recently held by the medical faculty assembled in Berlin.

AT the laying of the foundation-stone of the new Stanbridge Dispensary, it was stated that Mr. Corbett had given £12,000 for the Stanbridge and Brierley Infirmary. The dispensary was to be built with money bequeathed by Mr. Cole.

THE Women's Temperance League issued an especial manifesto exhorting the inhabitants of the metropolis to celebrate the Royal wedding in a sober manner, and condemned the practice of "drinking healths" in intoxicating liquors.

THE new Clarence wing of St. Mary's Hospital is in process of erection. At present the authorities are compelled to lodge part of the nursing staff out of the hospital. The wing, when completed, will have an extensive frontage to Praed Street.

AT a recent prosecution for selling adulterated milk in Oxfordshire, the defendant urged that the larger percentage of water in the milk was due to the drought, as the cows could not get sufficient nourishing food. He had, however, to pay the fine.

THE question of several adjacent districts combining to support an infectious diseases hospital was mentioned at the meeting of the South Stoneham Guardians recently, an infectious diseases hospital under present legislative conditions being necessary.

THE Pasteur Institute at New York has been removed to more commodeous quarters facing the Central Park. The building is six stories high and there is a superstructure of iron on the roof where animals which are used for obtaining virus will be kept.

ON the last day in the second week of June there were 89,972 paupers in receipt of relief. Besides these there were 824 vagrants who on the same day received temporary relief. These numbers show a considerable increase with corresponding numbers last year.

A LETTER in the *Morning Post* advocates dividing London into districts for the purpose of raising subscriptions for the voluntary hospitals, and suggests that each hospital should have a district assigned it and a committee to manage the collection of subscriptions.

SURGEON-GENERAL SIR WILLIAM MOORE, writing in the *Medical Magazine*, cites a number of striking facts relating to the dissemination of disease through flies. Sir William Moore has had exceptional opportunities of observing personally the extent of this evil in the East.

AT the 57th annual meeting at the Yarmouth Hospital, it was stated that the good work done by the hospital in the past year was in excess of that in previous years. The treasurer's report states that the invested funds now amount to £16,120; whilst the balance in hand is £400.

A GOOD many firms will await Dr. Nansen's verdict on the comparative excellence of their commodities. Bovril has been largely patronized. Messrs. Armour and Co., of Chicago, have provided a five years' supply of their meat products, and Messrs. Cadbury have sent 1,500 lbs. of their cocoa and chocolate.

THE annual trip to sea in aid of the Merchant Seamen's Orphan Asylum took place on July 1st. Six hundred ladies and gentlemen joined in the trip and a number of the girls' and boys' band from the asylum were present. A considerable sum of money was realized and handed over to the institution.

THE Croydon County Council have instructed the Infectious Diseases and Hospital Committee to empower the Town Clerk to employ counsel and professional witnesses to oppose any movement by the Metropolitan Asylums Board to set up an establishment for infectious diseases at Grange Wood, Upper Norwood.

AN influential committee has been formed, and subscriptions are being raised to protest against the proposed fever hospital at Upper Norwood. The hospital is intended to be established at Grange Wood, in a mansion standing in beautiful grounds near Beulah Hill. The ratepayers object to it as destroying the amenities of an important residential quarter.

THE Victoria Hospital for Sick Children at Hull has still a debt of £1,000 on the building fund. Mr. Arthur Wilson, who for many years has been a most generous friend of the hospital, has again given evidence of his interest in its welfare by offering a donation of £500 towards this object, subject to the remaining £500 being raised before the end of the year.

THE Medical Council of Leipsig passed a vote of congratulation on Dr. Ferdinand Leonhardi on the occasion of the 50th year of his practice amongst them. These were post mortem congratulations, for the doctor had been dead some years. This letter, strangely enough, went to a nephew of the deceased doctor, who himself had practised within a year of the same length of time.

### MEDICAL VACANCIES AND APPOINTMENTS.

### GUY'S HOSPITAL MEDICAL SCHOOL.

The following is a complete list of the medallists and prizemen presented to Sir John Lubbock at the distribution of prizes on the 17th inst.:—

THE TREASURER'S GOLD MEDAL FOR CLINICAL MEDICINE.—F. J. Colman, Hunmanby, Yorks.

THE TREASURER'S GOLD MEDAL FOR CLINICAL SURGERY.—G. Lawrence, Liverpool.

GURNEY HOARE PRIZE FOR CLINICAL STUDY.—C. C. Elliott, Wynberg, Cape Colony.

BEANEY PRIZE FOR PATHOLOGY.—F. J. Coleman, Hunmanby, Yorks.

GOLDING BIRD PRIZE AND GOLD MEDAL.—E. A. Peters, St. Leonards-on-Sea.

MICHAEL HARRIS PRIZE FOR ANATOMY.—W. E. Plummer, Tunbridge Wells.

THE ARTHUR DURHAM PRIZES FOR DISSECTION.—*First Year's Students*: R. P. Rowlands, Towyn, N. Wales, prize £5; J. O. Garland, Barnsley, certificate; E. I. Spriggs, Market Harborough, certificate. *Second Year's Students*: F. W. Rowland, Bromley, Kent, prize £15; W. J. Rowland, Bromley, Kent, certificate; A. G. Butler, Beckenham, certificate.

FOURTH YEAR'S PRIZES.—J. A. Howard, Canonbury, first prize, £25; G. S. Hovenden, South Norwood, second prize, £10.

THIRD YEAR'S PRIZES.—E. C. Taylor, Sydenham, first prize, £25; A. Salter, Blackheath, second prize, £10.

SECOND YEAR'S PRIZES.—F. J. Steward, Old Charlton, first prize, £25; C. J. Harnett, Sittingbourne, second prize, £10; H. A. Moffat, Grahamstown, Cape Colony, certificate; C. H. Fagge, Lutterworth, certificate; S. Copley, Pontypool, certificate; T. M. Thomas, Aberayron, Wales, certificate; P. N. Vellacott, West Thurrock, certificate. *Dental Students*: J. B. Parfitt, Reading, prize £15; F. C. Bromley, Southampton, certificate; E. Clayton, Wallington, certificate.

FIRST YEAR'S PRIZES.—W. C. Pakes, Garstang, first prize, £50; A. G. Butler, Beckenham, second prize, £25. *Dental Students*: H. Wallis, Hull, prize £10; U. E. Cave, Newbury, certificate: W. H. Pilcher, Godalming, certificate.

PRACTICAL DENTISTRY PRIZE.—E. V. Knight, Oxford, and J. B. Parfitt, Reading, equal, £5 each; W. R. Wood, Brighton, certificate; P. F. Henry, Peckham Rye, certificate.

OPEN SCHOLARSHIPS IN ARTS.—A. W. Nourse, Putney, 100 guineas, H. W. Bruce, Bow, 50 guineas.

OPEN SCHOLARSHIPS IN SCIENCE.—E. I. Spriggs, Market Harborough, 125 guineas; F. W. Goldie, Clapham Common, 50 guineas; R. T. Fitz-Hugh, Nottingham, certificate; H. J. Starling, Maida Hill, certificate.

### APPOINTMENTS.

F. W. Apthorp, L.R.C.P.Edin., M.R.C.S.Eng., appointed Medical Officer for the No. 5 District of the Cuckfield Union. Dr. W. J. T. Barker, appointed Medical Officer for the No. 2 (Streatham) District of the Wandsworth and Clapham Union. F. A. Barrington, L.R.C.P., L.M., L.R C.S.I., appointed Assistant Medical Officer of Health to the Lynn Town Council. G. G. Bothwell, M.B., C.M.Aberd., appointed Assistant House Surgeon to the Infirmary, Halifax. T. Brander, M.B., C.M. Aberd., appointed Medical Officer to the Lossiemouth Parochial Board. R. H. Browne, L.R.C.P.Lond., M.R.C.S.Eng., appointed Physician to the Chelsea, Brompton, and Belgrave Dispensary. T. L. Bryan, M.B.Durh., appointed Medical Officer for the Borough District of the East Ward Union. A. T. Drake, M.B., L.R.C.S.I., appointed Medical Officer for the No. 1 (Deptford) Sanitary District of the Greenwich Union. A. H. Godson, B.A., M.B., B.C.St. John's, Cambridge, and of Guy's Hospital, appointed House-Surgeon to the Croydon General Hospital. M. R. Gooding, L.R.C.P.Lond., M.R.C.S.Eng., appointed Medical Officer of Health to the Bideford Urban Sanitary District. W. H. Griffiths, M.R.C.S.Eng., L.S.A., reappointed Medical Officer of Health for the Hinckley Urban Sanitary District. C. Harwood, M.D., L.R.C.S.Edin., reappointed Medical Officer of Health for the Shardlow Rural Sanitary District. W. L. Hickey, M.D., B.Ch.Dub., appointed Medical Officer for the Gainsford District of the Teesdale Union. Dr. C. B. Hillyar, appointed Medical Officer for the Milton Abbot District of the Tavistock Union. H. Hodgson, M.R.C.S.Eng., L.R.C.P.Lond., appointed Medical Officer for the Blisworth District of the Towcester Union. A. A. Hogarth, M.B., C.M.Glasg., appointed

### MEDICAL APPOINTMENTS.

### ROYAL FREE HOSPITAL, Gray's Inn Road.

### CHARING CROSS HOSPITAL.

### RESIDENT MEDICAL OFFICER.

### MEDICAL VACANCIES AND APPOINTMENTS—(*continued*)

Medical Officer for the Third District of the Cheltenham Union. C. Husband, M.R.C.S.Eng., L.S.A., reappointed Medical Officer of Health for the Ripon City. J. Knott, M.D., B.Ch., M.R.C.P., F.R.C.S.I., appointed Medical Officer to the Society for the Prevention of Cruelty to Children, Dublin. T. C. Lawson, M.R.C.S.Eng., appointed Medical Officer for the Warboys District and Wistow Districts of the St. Ives Union. A. W. Leachman, M.D. St. And., M.R.C.S., D.P.H. Camb., appointed Medical Officer of Health for the Horsham Urban Sanitary District. J. Logie, M.B., C.M.Aberd., appointed Public Vaccinator for Woolwich.

### VACANCIES.

The following vacancies are announced this week, the amount of salary in each case being given after the name of the institution:—

#### Resident Medical Officers.

Asylum for Idiots, Earlswood, Redhill, Surrey. Assistant Medical Officer. Salary, £150 per annum, with board and residence. Application in envelope marked "Assistant Medical Officer," to the Secretary, 36, King William Street, London Bridge, E.C., by July 25th.

County Asylum, Lancaster. Assistant Medical Officer; unmarried. Salary £100 per annum, increasing £15 annually to £200 per annum, with furnished apartments, board, &c. Also Clinical Assistant. No salary; board, lodging, &c., provided. Applications to Dr. Cassidy, Medical Superintendent, by July 24th.

General Hospital for Sick Children, Pendlebury, Manchester. Junior Resident Medical Officer, doubly qualified. Salary £80 per annum, with board and lodging. Applications to the Chairman of the Weekly Board by July 29th.

Great Northern Central Hospital, Holloway Road, N. House Surgeon. Salary £60 per annum, with board and lodging in the hospital. Applications to W. F. Grant, Secretary, by July 24th.

Great Yarmouth Hospital. House Surgeon, doubly qualified. Salary, £90 per annum, with board and lodging. Applications to R. E. Ferrier, Hon. Secretary, by August 1st.

Hereford County and City Asylum. Locum tenens. £2 2s., with board and lodging. Application to the Medical Superintendent.

Hospital for Consumption and Diseases of the Chest, Brompton. Resident Medical Officer, doubly qualified, and not under 25 years of age. Salary £200 per annum, with board and residence. Applications to Hen. Dobbin, Secretary, by July 26th.

Liverpool Dispensaries. Assistant Surgeon; unmarried. Salary £80 per annum, with apartments, board, and attendance. Applications to R. R. Greene, Secretary, by July 24th.

Manchester Royal Infirmary. Resident Medical Officer, not less than 25 years of age and unmarried; doubly qualified. Appointment for one year, but holder of office may be re-elected. Salary £150 per annum, with board and residence. Applications to the Chairman of the Board by July 29th.

Nottingham and Borough Asylum. Second Assistant Medical Officer, unmarried. Salary £100 per annum, with apartments, board, and washing. Applications to the Medical Superintendent by July 25th.

Royal Portsmouth, Portsea, and Gosport Hospital. Assistant House Surgeon; appointment for six months. Board, residence, and washing, and an honorarium of £15 15s. Applications to J. A. Byerley, Secretary, 137, Queen Street, Portsea, by July 27th.

Royal South Hants Infirmary, Southampton. Assistant House Surgeon. No salary; board and rooms provided. Applications to T. A. Fisher Hall, Secretary, by July 31st.

Stafford General Infirmary, Stafford. Assistant House Surgeon. Board and residence provided. Applications to the House Surgeon by July 20th.

Stockport Infirmary. Junior Assistant Surgeon; appointment for six months. Board and residence provided, and an honorarium of £10 after satisfactory service. Applications to Lieutenant-Colonel S. W. Wilkinson, Honorary Secretary, by July 25th.

Whitehaven and West Cumberland Infirmary, Whitehaven. House Surgeon; doubly qualified. Salary £120 per annum, and £30 per year for dispensing, with furnished apartments and attendance. Applications to Tyson Kitchen, Secretary, by July 29th.

#### Non-Resident Medical Officers.

Charing Cross Hospital, W.C. Assistant Surgeon: must be F.R.C.S. Eng. Applications to the Chairman of Committee by July 24th.

Hospital for Sick Children, Great Ormond Street, Bloomsbury, W.C. Assistant House Surgeon; appointment for six months. Salary £20. Applications to the Secretary by July 26th.

Kidderminster Union. Medical Officer for the Bewdley District. Salary £100 per annum and vaccination fees. Applications to Mr. F. Burcher, Clerk, Workhouse, Braintree, by July 24th.

Pontypridd Rural Sanitary Authority. Medical Officer of Health. Salary £50 per annum. Applications to E. C. Spickett, Clerk, by July 27th.

Royal Free Hospital, Gray's Inn Road, W.C. Assistant Physician. Applications to the Secretary by July 31st.

The Yorkshire College, Leeds. Demonstrator of Anatomy. Particulars from the Registrar.

Wolverhampton and Staffordshire General Hospital, Wolverhampton. Honorary Gynæcologist; doubly qualified. Applications to the Chairman of the Weekly Board by July 24th.

